# Effect of electroacupuncture versus solifenacin for moderate and severe overactive bladder: a multi-centre, randomized controlled trial study protocol

**DOI:** 10.1186/s12906-020-03018-y

**Published:** 2020-07-16

**Authors:** Qian Wen, Ning Li, Xueling Wang, Hao Li, Fengwei Tian, Weiwei Chen, Yanyan Lu, Zhishun Liu

**Affiliations:** 1 Department of Integrated Traditional and Western Medicine, West China Hospital, Sichuan University, Chengdu, China; 2 Department of acupuncture and moxibustion, Chongqing hospital of traditional Chinese medicine, Chongqing, China; 3 Department of acupuncture and moxibustion, Subei people’s hospital of Jiangsu province, Yangzhou, China; 4 Department of acupuncture and moxibustion, Jiangbin hospital of Guangxi Zhuang autonomous region, Nanning, China; 5 Guang’anmen Hospital, China Academy of Chinese Medical Sciences, No.5 Beixiange Street, Beijing, Xicheng District China

**Keywords:** Electroacupuncture, Solifenacin, Overactive bladder, Randomized controlled trial

## Abstract

**Background:**

Overactive bladder is defined as “urgency, with or without urge incontinence, usually with frequency and nocturia”. Electroacupuncture may be a safe and an effective alternative therapy for overactive bladder, but the evidence is limited.

**Methods:**

We will conduct a three-arm, non-inferiority, multi-centre randomized controlled clinical trial. A total of 420 patients with moderate and severe overactive bladder will be randomly assigned to one of three groups: the electroacupuncture group (*N* = 140), sham electroacupuncture group (*N* = 140), and solifenacin group (*N* = 140). The primary outcome will be the change in the overactive bladder symptom score from baseline to the end of the 12-week treatment. The secondary outcomes will include the proportion of participants with a decrease in the overactive bladder symptom score ≥ 3 at weeks 4, 8, 12, 20, and 32; the change in average 24 h values of urination, nocturnal urination, urgency incontinence and urgency episodes from baseline to weeks 4, 8, 12, 20 and 32, and so forth. The adverse events will be recorded. Statistical analysis will include covariance analysis, nonparametric tests and descriptive statistics.

**Discussion:**

This study will answer the question of whether electroacupuncture is effective and non-inferior to solifenacin for improving the symptoms of overactive bladder patients.

**Trial registration:**

Chinese clinical trial registry (ChiCTR1800019928).

## Background

Overactive bladder, also known as OAB, or overactive bladder syndrome, is defined as “characterized by urinary urgency, with or without urgency urinary incontinence, usually with increased daytime frequency and nocturia, if there is no proven infection or other obvious pathology.” by the International Continence Society (ICS) [1]. The detrusor can be considered hyperactive on urodynamics in some patients. The guidelines for the diagnosis and treatment of OAB in adults were first presented by the American Urological Association (AUA) in 2012 [2] and updated in 2015, where the definition of OAB is consistent with that of ICS but emphasizes the exclusion of neurogenic bladder [3]. According to the first large population-based study of OAB conducted in six European countries using the 2002 ICS definitions, the prevalence of OAB was 11.8% (10.8% men; 12.8% women) among people aged 40–64 years [4]. China’s first large-scale epidemiological survey of OAB showed that for people over 18 years old, the overall prevalence was 6% and was 11.3% for people over 50 years old, which increasing with age [5]. OAB, with a severely negative impact on patients’ mood and health, increases both economic and social burden [6].

The latest guidance from the AUA in 2015 classifies OAB treatment regimens into first-line, second-line, third-line and others. The first-line treatment consists of behavioural modifications, such as bladder training, bladder control strategies, pelvic floor muscle training, and fluid management. Second-line treatment consists of pharmacologic management, mainly drugs are antimuscarinic (AM) agents and β3-adrenoceptor agonists. Solifenacin is an AM, especially a specific competitive inhibitor of the M3 subtype, and is one of the most commonly used drugs for the treatment of frequent urination, urgent urination, nocturia and urgency urinary incontinence in patients with OAB [7]. Intramuscular injection of botulinum toxin type A into the detrusor muscle, percutaneous tibial nerve stimulation (PTNS), and sacral neuromodulation (SNS) are types of third-line treatments. The other treatment is surgery [3].

Behavioural modifications pose few risks to patients, but the efficacy depends on the positivity and persistence of patients [8]. As second-line treatments, antimuscarinic agents can lead to many adverse effects, including dry mouth, blurred vision, constipation, and so on. And the tachycardia, hypertension, headache and other adverse reactions may occur with the beta-3-receptor agonists. Inefficacy and adverse events are the most common reasons for the withdrawal of AM [9]. A retrospective cohort analysis indicated that adherence rates to AM ranged from 27.6 to 40.4% over 12 months [10]. In terms of SNS, a systematic review [11] showed that this treatment could significantly reduce the episodes of urination and urinary incontinence and could reduce the use of pads; however, the complications after implantation include pain at the stimulation site, infection and changes in intestinal function. PTNS is the other treatment for OAB that has been confirmed to improve symptoms [12].

As a main branch of traditional Chinese medicine, acupuncture has been recommended for solving neurogenic bladder dysfunction by the World Health Organization [13]. Our team’s latest OAB system evaluation suggests that electroacupuncture (EA) may reduce urinary frequency, urinary incontinence, and nocturnal urination episodes [14]. However, the evidence does not support the efficacy of EA alone in the treatment of OAB. In the future, high-quality research methods and large sample sizes are needed to confirm the efficacy of EA in OAB. EA is similar to PTNS in some ways; however, the type of EA treatment used in this trial could stimulate not only the tibial nerve (through the acupoint of SP6) but also the 3 sacral (through the acupoint of BL33) and pudendal nerves (through the acupoint of BL35). Integrating PTNS and SNS stimulation points may have good benefits. Some research has been conducted to prove the effectiveness of EA for the treatment of OAB. A study conducted by LU Yujin et al. included 86 OAB patients with EA, and the results showed an overactive bladder symptom score (OABSS) of 8 (CI: 7–9) before treatment and 2 (CI: 4–6) after treatment, with a statistically significant difference (*P* < 0.05) [15]. A randomized controlled trial (RCT) conducted by Wang Yafei et al. suggested that EA had significant clinical efficacy for OAB with a simple operation, and there were no side effects or postoperative complications, which was better than the medicine [16]. Another animal experiment proved that EA could effectively inhibit bladder overactivity, and the effect had acupoint specificity [17].

Thus, based on the aforementioned historical study and clinical experience, our team designed this prospective, multi-centre, RCT study protocol with a large sample size to evaluate the effectiveness and safety of EA compared with solifenacin for OAB.

## Methods and design

### Aims, design, and study setting

#### Primary objective and study hypothesis

The primary objective is to assess the effect of EA on the treatment of OAB. We hypothesize that EA will be effective and non-inferior to solifenacin for improving the symptoms of OAB patients.

#### Study design

**Fig. 1:**
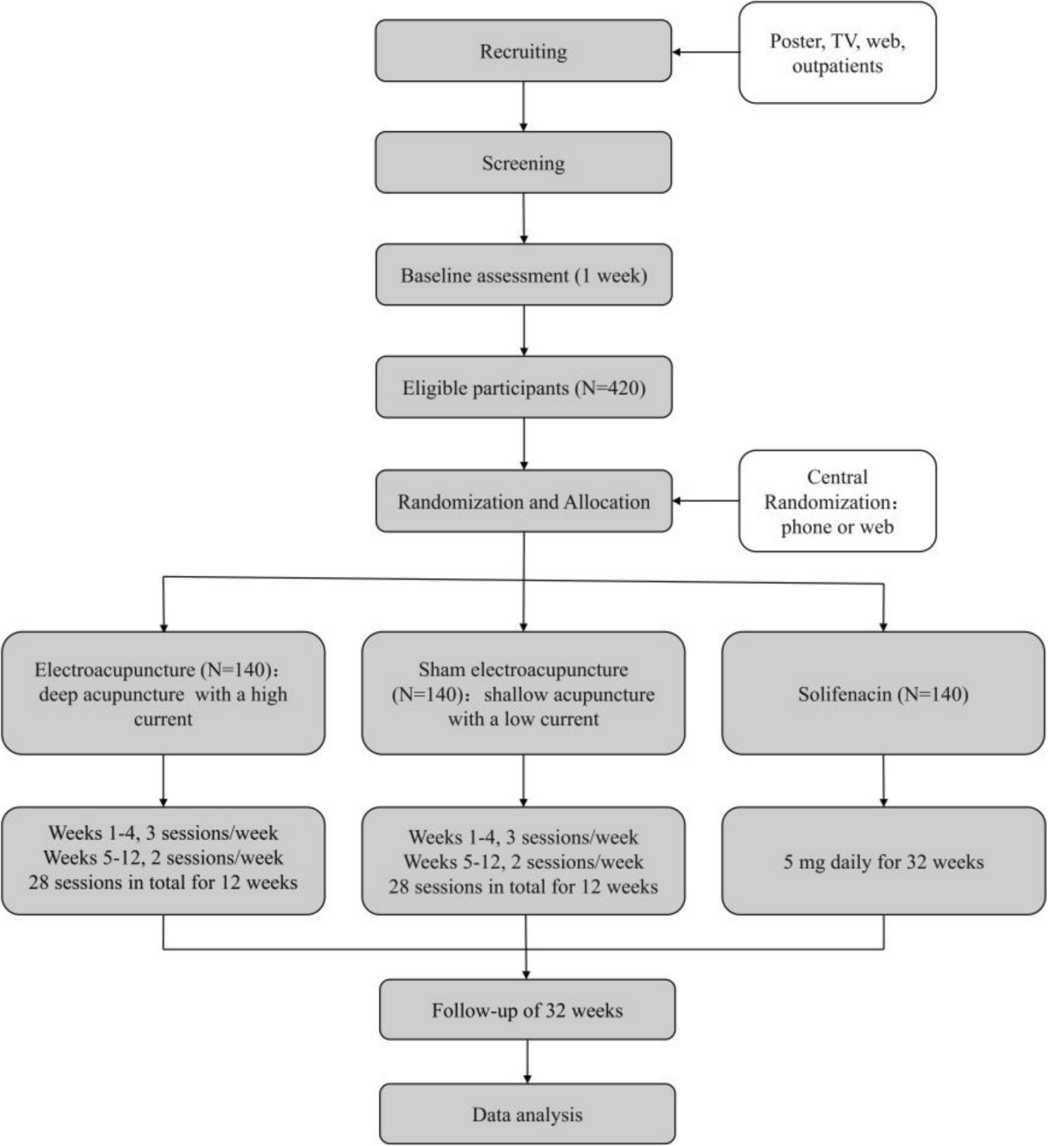
The flowchart

This study will be a multi-centre, randomized, parallel group, and three-arm clinical trial. It will be conducted from September 2019 to June 2021 in 4 hospitals around China. If the study is not expected to be completed on time, 2 research hospitals will be added or replaced. Participants with OAB will be recruited via posters, television, websites and outpatients and will then be screened according to the eligibility criteria. A total of 420 participants will be ultimately enrolled. Prior to enrolment, all participants will voluntarily sign the informed consent form that has been approved by the ethics committee. Randomization will be performed centrally by the Clinical Evaluation Centre of the China Academy of Chinese Medical Sciences in Beijing, and eligible participants will be randomized to the EA group, sham electroacupuncture (SA) group or solifenacin group at a 1:1:1 allocation ratio. A randomization number will be applied after enrolment. The statisticians, outcome assessors, and participants in the EA and SA groups will be blinded to the allocation. A flowchart demonstrating an outline of the study is included in Fig. 1.

#### Study setting

This study will be conducted at the West China Hospital of Sichuan University, Chongqing Hospital of Traditional Chinese Medicine, Subei People’s Hospital of Jiangsu Province, and Jiangbin Hospital of Guangxi Zhuang Autonomous Region. Among them, three are Western medicine hospitals and one is a traditional Chinese medicine hospital.

### Ethics

This trial protocol (version no.20180821, date 9th October,2018) is in accordance with the ethical standards described in the 2013 updated Declaration of Helsinki [18]. All research units have been reviewed by the West China Hospital of Sichuan University clinical trials and biomedical ethics committee before the start of the clinical study (the ethical approval number is 2018–386). The study has also been registered at http://www.chictr.org.cn (ChiCTR1800019928). The study will be reported in compliance with the CONSORT statement (www.consort-statement.org) as well as the standards for reporting interventions in clinical trials of acupuncture (STRICTA) [19, 20].

### Eligibility criteria

#### Participants

The included participants will be required to meet the eligibility criteria. Participants will be evaluated by professional researchers. For participants who meet the inclusion criteria, the researcher will discuss the informed consent form and explain the risks and advantages of participating in the study in detail. Written informed consent will be obtained prior to the participants’ enrolment. Then, the researcher will again check whether the participants meet the inclusion criteria, and the confirmed participants will be randomly assigned to the EA group, the SA group or the solifenacin group.

#### Inclusion criteria

Patients with OAB who meet all of the following will be enrolled in the trial:

1. Meet the diagnostic criteria of the ICS and AUA for OAB [1–3];
2. Aged 18–75 years;
3. Have suffered from OAB ≥3 months;
4. Have an OABSS ≥6;
5. Are voluntarily willing to sign informed consent.

#### Exclusion criteria

Patients who meet any of the following criteria will be excluded from the trial:

1. Frequency and urgency of urination occur secondary to other diseases (secondary OAB);
2. Mixed urinary incontinence patients;
3. Patients with uncontrolled symptomatic urinary tract infection;
4. Pelvic organ prolapse ≥ degree II;
5. Bladder urine residue ≥100 ml;
6. Allergy to metal, fear of acupuncture, or cannot tolerate EA therapy;
7. Allergy to solifenacin or have anti-muscarinic contraindications, such as urinary retention, gastrointestinal retention, myasthenia gravis, ulcerative colitis, and angle-closure glaucoma;
8. Uncontrolled diabetes; severe heart, liver, kidney, or mental illness; or coagulopathy;
9. Patients with a pacemaker;
10. Pregnancy or lactation period or have pregnancy intentions in the next year;
11. Drug treatment or acupuncture for OAB within 1 month;
12. Participants in other research projects.

#### Withdrawal criteria

The withdrawal criteria will be as follows:

1. Severe side effects assessed by a urologist at each centre;
2. Serious complications or other serious diseases during the study;
3. Withdrawal consent by the patient.

### Randomization and blinding

After the 1-week assessment has been carried out and informed consent has been obtained, participants will be divided into the EA group, SA group or solifenacin group at a ratio of 1:1:1 due to the completely randomized block design of the hospital factor. Randomization will be generated by the central random system based on the computer network. Randomized numbers and groupings will be applied by research assistants who are not involved in the treatment or evaluation. Patients in the EA group and the SA group will be blinded, while those in the solifenacin group will be subjected to open trials. In the informed consent signed before the participants were enrolled, we will inform them that there are two types of acupuncture in addition to the drug group, and participants will be required to complete the entire trial cycle no matter which group they are assigned to. After the last treatment, the evaluation for the blinding of acupuncture will be tested within 5 min in the EA and SA groups. The results of this evaluation will be used to analyze the effect of blinding on the trial outcome measures. Efficacy evaluation and data entry will be performed by research assistants who are not involved in the grouping. The statisticians, outcome assessors, and physicians will be blinded to the allocation during data summarization.

### Procedure

After randomization, therapeutic assessment will be conducted at six time points: baseline and at the end of weeks 4, 8, 12, 20, and 32. Full-time evaluators will be responsible for filling out, issuing, recovering and recording the urination diary card and case report form (CRF) during the trial.

### Intervention

Participants randomized to the EA group will receive a standardized 30-min EA treatment; the SA group is similar to the EA group except in terms of the current, the size of the needle, and the depth of the needle insertion; the patients in the solifenacin group will be treated strictly following pharmaceutical guidelines. All acupuncture treatment sessions will be performed by Chinese medicine practitioners registered as acupuncturists and who have at least 2 years of clinical experience in acupuncture practice. No participants will receive any other treatments.

### Electroacupuncture (EA)

According to traditional Chinese medicine theory, the results of previous studies and the opinions of acupuncture experts at Guang’anmen Hospital, China Academy of Chinese Medical Sciences, the following main acupoints will be selected: BL23 (Shen Shu), BL33 (Zhong Liao), BL35 (Hui Yang), and SP6 (San YinJiao). All acupoints are bilateral. The locations of each acupoint conform to the standard defined by the World Health Organization [21].

After sterilizing the acupoint areas, for BL33, a needle will be inserted at a point 1 cm upwards and outwards from the 3rd posterior sacral foramina into the 3rd posterior sacral foramina in a slant and in an inward and a downward direction as well as at an angle of approximately 45–70 degrees to the midline using a 0.30 mm × 75 mm needle inserted approximately 60–70 mm deep. For BL35, a needle will be inserted (needle size 0.30 mm × 75 mm) approximately 60–70 mm deep at a slant in an outward and upward direction. For BL23 and SP6, needles will be directly inserted approximately 25–30 mm deep with 0.30 mm × 40 mm needles. The duration of reinforcing- reducing manipulation of twirling and rotating needle should be used for 1 min to achieve de qi (a composite of sensations including acid, hemp, bilge sensation, warm and pulse, these feelings may even travel along the meridians), which is recognised to be an essential component for acupuncture efficacy. Then electrodes from the EA apparatus will be attached transversely to the needle handles for bilateral BL33 and BL35 with a current intensity of 2 to 6 mA and with a current intensity of 1 to 2 mA for ipsilateral BL23 and SP6. All current intensities will be as high as can be tolerated. The current frequency will be a continuous wave of 10 Hz, and the stimulation will last for 30 min. First, EA will be applied for three sessions per week for a total of 4 weeks, followed by twice a week for 8 weeks a total of 28 sessions over a course of 12 weeks.

### Sham electroacupuncture (SA)

The non-acupoints will be located 2 cm outward for BL33, BL35, BL23 and SP6. After local skin disinfection, a 0.2 mm × 25 mm needle will be used for insertion at a depth of approximately 2–3 mm until the needle can stand erectly. The manipulation of the needle to de qi will not execute, and the current intensity will be 0.1–0.3 mA (slight electrical stimulation is perceived by the patient). The connection method for the electrode and the number of sessions of acupuncture will be the same as those for the EA group.

### Solifenacin

Solifenacin will be taken orally at a dose of 5 mg once daily for 32 weeks. It will be suggested that patients who can tolerate the drug should continue to take the drug; the drug should be withdrawn in cases of failure; and patients with severe adverse reactions should stop taking the medicine at any time, which will be recorded.

### Outcome measures

In this study, there will be one primary outcome and nine secondary outcomes. These are presented in Table 1. All adverse events will be recorded. The OABSS is widely used as a primary outcome measure in OAB studies, as it is safe and convenient and has been proven to have good reliability and validity in OAB [22]. The OABSS is comprised of 4 test items including frequency of urination, nocturia, urgency urinary incontinence and urgency episodes in 24 h. Total score ranges from 0 to 15, with scores of 3 to 5 indicating mild OAB, 6 to 11 indicating moderate OAB, and 12–15 indicating severe OAB. The secondary outcomes are based on 7-day bladder diaries and the OAB-q. A previous study suggested that 7-day bladder diary provided more accurate information regarding incontinence than a 3-day diary [23], in which we will evaluate the average episodes of 24 h urination, nocturia, urge incontinence and urgent urination changes from baseline. The OAB-q, with 33 questions about sleeping, anxiety, trouble in life and so on, is appropriate for assessing symptomatic distress or assessing the impact of OAB on health-related quality of life [24].

**Table 1:** Outcome measures

	Outcome measure	Time point	Description	Statistics
Primary outcome	Change in OABSS from baseline to the end of week 12	Week 12	Calculated based on the OABSS.	Modified t-test
Secondary outcomes	Change in OABSS from baseline to the end of weeks 4, 8, 20, and 32	Weeks 4, 8, 20, and 32	Calculated in the same way as the primary outcome.	Covariance analysis or non-parametric test
	Proportion of participants with a decrease in the OABSS of ≥3	Weeks 4, 8, 12, 20, and 32	The number of patients with a reduction in OABSS ≥3 divided by the number of participants at baseline.	A chi-square test or Fisher’s test
	Change in 24 h urination/nocturia/urgency urinary incontinence /urgency episodes from baseline	Weeks 4, 8, 12, 20, and 32	The average 24 h urination/nocturia/urgency urinary incontinence /urgency episodes equal to the sum of urination/nocturia/urgency urinary incontinence/urgency episodes divided by 7.	Covariance analysis or non-parametric test
	Change in OABSS for each question from baseline	Weeks 4, 8, 12, 20, and 32	Calculated based on the OABSS for each question.	Covariance analysis or Non-parametric test
	Change in OAB-q from baseline	Weeks 4, 8, 12, 20, and 32	Calculated based on the OAB-q.	Covariance analysis or non-parametric test
	Descriptive analysis of emergency medications and other treatments and drugs used at baseline, during therapy and at follow-up.		A questionnaire will be completed by participants to collect information regarding their other treatments.	Descriptive statistics
	Evaluation of the blinding of acupuncture	Week 12	After the last treatment, the evaluation for the blinding of acupuncture will be tested within 5 min in the EA and SA groups	Descriptive statistics
	EA acceptance assessment	Week 12	After the last treatment, the acceptance of EA will be tested within 5 min with a 5-point scale (‘1’ indicates easily accept and ‘5’ indicates very difficult to accept). Average scores will be calculated.	A chi-square test or Fisher’s test
	Evaluation of the intervention expectation	Weeks 0 and 12	A questionnaire will be completed by participants to evaluate their expectations of the intervention.	A chi-square test or Fisher’s test

### Safety evaluation

All adverse events will be recorded on the adverse event record sheet by the acupuncturist and participants at any time over the study period. Adverse events include broken needle, unbearable acupuncture pain, local haematoma, infection, dry mouth, constipation and any other discomfort or accident. The intensity and causality of each adverse event will be evaluated and recorded. If any serious adverse events occur as the result of a certain intervention, that intervention will be stopped immediately, and appropriate corrective action will be taken. Any serious adverse events will be reported promptly to the institutional review board within 24 h until 30 days after the end of the trial. If any of the enrolled patients become pregnant, it will also be reported by filling in the “pregnancy notice form” to give to the institutional review board.

### Quality control

A central randomization system will be adopted, which will be undertaken by the China Academy of Chinese Medicine Sciences to reduce selection bias. Before starting the trial, all researchers from the 4 trial centres will undergo training. All collected data will be timely input into the ClinResearch Electronic Data Capture System developed by the Clinical Evaluation Center of the China academy of Chinese Medical Sciences. A 3-level monitoring system will be established to periodically check the performance of the trial. The outcome assessments and the completion of CRF and data management will be closely supervised.

### Sample size calculation

The sample size will be calculated based on previous studies conducted by Zhao Y et al. [14, 25]. A sample size of 420 patients will provide 80% power (α = 0.05) to detect a non-inferiority effect of EA therapy on the primary outcome, assuming a 10% loss. The non-inferiority margin will be 50% of the comparative efficacy between SA and solifenacin. The non-inferiority margin will be set based on our hypothesis that EA will be associated with at least 50% of the difference in efficacy of solifenacin compared with SA.

### Measures to improve compliance

Establishing a good relationship between doctors and patients, and providing participants with subsidies for transportation costs. A patient manual for regular short message services will be used to remind patients to complete the bladder diary.

### Statistical analysis

Statistical analysis will be conducted by independent third-party professional statisticians. We will use a modified t-test based on the retention of the effect method to analyse whether EA is non-inferior to solifenacin [26]. If the lower boundary of the confidence interval around the mean difference between the numbers in the EA group versus the solifenacin group exceed 50% of the mean difference between the numbers in the solifenacin group and the SA group, then non-inferiority will be claimed. The intention-to-treat (ITT, randomized participants) and per-protocol datasets (PP, ITT participants who received at least one treatment session) will be used to analyse the primary outcome. Missing data will be filled with a multiple imputation method under the missing-at-random assumption. Secondary outcomes will be calculated with observed values. Categorical data will be described by case number and percentage and will be analysed with Fisher’s exact test or the Chi-square test where appropriate. Continuous data will be described as the mean ± standard deviation, median, maximum and minimum values, and comparisons between groups will be conducted by covariance analysis or nonparametric tests where appropriate. A P (2-tailed) value less than or equal to 0.05 will be considered statistically significant. Statistical Package for the Social Sciences (version 13.0; SPSS; Chicago, Illinois, USA) will be used to perform the statistical analysis.

## Discussion

OAB is a common disease among people in many countries. The economic burden in terms of managing OAB is substantial according to research conducted in the following six countries: Canada, Germany, Italy, Spain, Sweden and the UK [27]. Acupuncture is a traditional Chinese medicine therapy that has been proven effective for lower urinary tract dysfunction with advantages of minimal adverse effects, being non-toxic, having a low socioeconomic burden [16, 28]. However, there have been few appropriately designed randomized control trials that have provided strong supporting evidence about the effectiveness of acupuncture for the treatment of OAB worldwide. Thus, in this non-inferiority, randomized controlled trial, we intend to compare the efficacy of EA versus solifenacin to evaluate whether EA treatment is effective in the management of OAB and its clinical value. If the effect of EA is non-inferior to that of solifenacin, it may be a reasonable option in patients with OAB as a complementary and alternative approach.

To assure the reproducibility of the study, we have carefully designed this RCT and have included thorough details about the plan for implementation, aiming to offer trustworthy evidence for the effects of EA on OAB. Computer-generated randomization and central randomized allocation concealment will be adopted to minimize selection bias, and blocked randomization will be applied to ensure prognostic balance between groups. Those outcome assessors, data managers and supervising staff will be blinded to the group allocation.

One of the limitations of this trial design is the non-blinding of the EA group and solifenacin group. Due to the characteristics of acupuncture and the drug, it is impossible to blind the participants and acupuncturists. The other limitation is the use of SA as a control in this trial. Because no ideal placebo-acupuncture exists [29], choosing an appropriate sham acupuncture as a control is important. In this trial, minimal use of EA for non-acupoints will be used as the SA control. In order to blind the patients successfully and the convinience of the operation, all patients will receive acupuncture with prone position, and expose the waist, hips and double lower limbs on the bed curtained off. They will not be able to see the differences of manipulation. During acupuncture, patients will be required to have quiet rest to avoid talking to each other. Doctors will give patients uniform instructions to reduce the impact of psychological suggestions on the efficacy of acupuncture.

In conclusion, under strict quality control, we expect that the results of this study will provide high-grade evidence to determine whether the effect of EA is non-inferior to solifenacin for improving the symptoms of patients with OAB.

### Trial status

This study is currently ongoing. The study commenced on September 2019, 23 participants have been recruited, and the anticipated end date is 30 June 2021.

## Data Availability

The datasets used and/or analysed after completing the current study will be available from the corresponding author upon reasonable request.

## Abbreviations

OAB: Overactive bladder
ICS: International Continence Society
AUA: American Urological Association
AM: Antagonists selective for receptor M
PTNS: Percutaneous tibial nerve stimulation
SNS: Sacral nerve stimulation
EA: Electroacupuncture
OABSS: Overactive bladder symptom score
CI: Confidence interval
RCT: Randomized controlled trial
SA: Sham electroacupuncture
STRICTA: Standards for reporting interventions in clinical trials of Acupuncture
CRF: Case report form
OAB-q: Overactive bladder questionnaire
ITT: Intention-to-treat
PP: Per-protocol
